# Clinical Perception and Treatment Options for Behavioral and Psychological Symptoms of Dementia (BPSD) in Italy

**DOI:** 10.3389/fpsyt.2022.843088

**Published:** 2022-04-01

**Authors:** Fabrizia D'Antonio, Lucio Tremolizzo, Marta Zuffi, Simone Pomati, Elisabetta Farina, Sindem BPSD Study Group, Margherita Alberoni

**Affiliations:** ^1^Department of Human Neuroscience, Sapienza University of Rome, Rome, Italy; ^2^Neurology “San Gerardo” Hospital Monza and University of Milano-Bicocca, Milan, Italy; ^3^Neurology Department, MultiMedica Castellanza, Milan, Italy; ^4^Centro per il Trattamento e lo Studio dei Disturbi Cognitivi, Ospedale Luigi Sacco, Milan, Italy; ^5^IRCCS Fondazione Don Carlo Gnocchi ONLUS, Milan, Italy

**Keywords:** BPSD (behavioral and psychological symptoms in dementia), psychosis, BPSD management, dementia, apathy

## Abstract

**Background:**

Behavioral and psychological symptoms of dementia (BPSD) have a high prevalence, and their presence is associated with a severe impact in terms of social costs. However, dedicated clinical tools or biomarkers to detect these symptoms are lacking. Thus, BPSD management in clinical settings is challenging. The aim of this study was to investigate the perception and the treatment strategies for BPSD in Italian centers working in the dementia field.

**Methods:**

A multicenter, national survey was developed by BPSD Study Group of the Italian Neurological Society for Dementia (SINDEM). The survey consisted of a semi-structured questionnaire that was e-mailed to SINDEM members, dementia centers part of the national network of memory clinics (Centers for Cognitive Deterioration and Dementia [CDCD]), and clinicians working in dementia care settings. The questions were focused on (1) perceived global frequency and relevance of BPSD; (2) tools used to assess BPSD; (3) pharmacological treatment for psychosis, apathy, agitation, aggression, depression, anxiety, sleep, and nutrition disturbances; (4) non-pharmacological treatments; (5) drugs side effects.

**Results:**

One-hundred and thirty-six clinicians participated in this study. Seventy-nine participants worked in a CDCD and 57 in other settings. The perceived frequency of BPSD was 74%. BPSD are detected by means of a clinical assessment for 96.3% or a caregiver interview for 97%. For psychosis treatment the first choice was atypical antipsychotics (83.3%), followed by typical antipsychotic (8.9%) and antidepressants (4.8%). For agitation, atypical antipsychotics were the first-choice treatment in 64% of cases and antidepressants in 16.1%. For aggression, the most used drugs were atypical antipsychotics (82.9%). For anxiety, 55.2% use antidepressants, 17.9% use atypical antipsychotics, and 16.9% use benzodiazepines. Interestingly, most of the centers apply non-pharmacological treatments for BPSD. Some differences emerged comparing the responses from CDCD and other care settings.

**Conclusion:**

The survey results revealed many differences in BPSD perception, treatment options, and observed side effect according to the clinical setting. This variability can be explained by the absence of clear guidelines, by differences in patients' characteristics, and by clinical practice based on subjective experience. These results suggest that producing guidelines for the pharmacological treatment of BPSD is a major need.

## Introduction

According to the original definition, behavioral, and psychological symptoms of dementia (BPSD) are “a heterogeneous set of psychological reactions, psychiatric symptoms and anomalous behaviors that appear in patients with dementia, of any etiology” (1). BPSD have been grouped in different clusters according with the characteristics of the examined populations or based on the settings in which subjects were evaluated. In a large cohort of newly diagnosed untreated patients with Alzheimer's disease (AD), evaluated with a 10-items version of the Neuropsychiatric Inventory (NPI) (2), a factor analysis identified five BPSD clusters, i.e., psychosis (delusions and hallucinations), affective syndrome (anxiety and depression), apathy, psychomotor syndrome (irritability, aberrant motor behavior, and agitation), and mania (euphoria and dis-inhibition). A longitudinal study focusing on BPSD in AD reported three clusters, i.e., psychotic syndrome (hallucinations, delusions), affective syndrome (depression, anxiety, irritability, agitation), and a behavior syndrome (euphoria, dis-inhibition, apathy, aberrant motor behavior) (3). In another study conducted in institutionalized non-demented elderly with the full 12-items NPI, neuropsychiatric clusters were defined in a slightly different fashion: affective (depression, anxiety, night-time behaviors), hyperactive (agitation, irritability, appetite abnormalities), psychotic (delusions and hallucinations), manic (euphoria and dis-inhibition), and apathetic (apathy and aberrant motor behavior) syndromes (4). A systematic review of the studies on BPSD in AD highlighted that the most inconsistent results for BPSD syndrome clustering concerned “sleep and nighttime behavior disorders” and “appetite and eating disorders” (5). These differences may depend on some factors such as age, severity of disease, heterogeneity of subjects' characteristics, and pharmacological treatments.

Some BPSD are part of the diagnostic core criteria for different forms of dementia (6, 7), and they can also appear before cognitive symptoms and overt dementia. In this case BPSD can be considered prodromal symptoms of dementia, according to the definition of Mild Behavioral Impairment (MBI), a condition which consists of the occurrence of BPSD in subjects without cognitive impairment or in patients with mild cognitive impairment (MCI) (8). Furthermore, specific neuropsychiatric syndromes can predict cognitive and functional decline progression in MCI patients with BPSD: manic syndrome has been found to be associated with a higher risk of cognitive decline, whereas affective syndrome has been found to predict functional decline (9).

BPSD prevalence is high and their presence is associated with severe caregivers' burden and a greater rate of institutionalization with a major impact in terms of social costs (10–13).

Despite their high prevalence, there are only a few dedicated clinical tools (scales/questionnaires) or biomarkers to detect these symptoms, and this may alter the BPSD perceived prevalence. Criteria have been established for apathy, depression, and psychosis, mainly in AD (14–17), with the aim to facilitate the enrollment in clinical trials.

Moreover, little is known about the neurobiological basis of BPSD and research on specific biomarkers associated with BPSD is lacking. Consequently, treatments tailored for BPSD do not exist. Also, in the scientific community no agreement has been reached on practice recommendations for BPSD, although non-pharmacological treatments are indicated as first-line, whereas pharmacological treatment are proposed as second-line options (18).

In clinical settings the management of BPSD is challenging and requires experience in the field and large competencies (19, 20). In this wide blunt context regarding BPSD diagnosis, detection, and treatment, it would be important to better characterize BPSD and harmonize the clinical and instrumental tools dedicated to these symptoms. As a first step to attain this goal, in this study the BPSD Study Group of the Italian Neurological Society for Dementia (SINDEM) aimed at investigating the clinical perception and the adopted treatment strategies for BPSD in Italian centers working in the dementia field.

## Materials and Methods

A multicenter national survey was developed by the BPSD Study Group of the Italian Neurological Society for Dementia (SINDEM). The survey consisted of a semi-structured questionnaire that was e-mailed to SINDEM members and dementia centers part of the national network of memory clinics, (Centers for Cognitive Deterioration and Dementia [Centri per i Disturbi Cognitivi e Demenze, CDCD]). To extend the number of participants, we also asked colleagues to email the questionnaire to other neurologists or geriatricians involved in dementia care in different settings. In the survey, the participants were asked to specify in which type of setting they worked. The questionnaire included either closed (yes/no) or multiple-choice responses. The questions were focused on (1) perceived global frequency and relevance of BPSD; (2) tools used to assess BPSD; (3) pharmacological treatment for psychosis, apathy, agitation, aggression, depression, anxiety, sleep, and eating disturbances; (4) non-pharmacological treatments; and (5) drugs side effects. In some questions participants were asked to order by frequency the observed symptoms or the drugs used to treat the symptoms. Multiple-choice questions investigated what techniques or biomarkers were used in clinical practice to investigate BPSD, including structural (CT or MRI) or functional neuroimaging (FDG-PET), along with genetic, blood, and cerebrospinal fluid (CSF) analyses. Finally, the participants were asked whether they could be interested in new scales and biomarkers for BPSD. The questionnaire answers were anonymous to let responders feel free to express their opinions. However, an email was sent to possible responders, to leave them the opportunity to declare their participation in the survey. This was done in order to allow participants to disclaim their interest in initiatives of the BPSD SINdem study group and to express suggestions and comments for a possible follow-up of the study. Among the 56 responders who disclaimed their participation, 49 were neurologists, 5 geriatricians, 1 psychiatrist, 1 was both neurologist and psychiatrist, and 2 were psychologists. Their experience ranged from 5 to 40 years (mean and standard deviation: 24.4 ± 11). Thirty-four had already performed some kind of research about BPSD and most of them were aware of BPSD guidelines. The questionnaire responses were first analyzed globally and then separately for responders working in CDCD and those working in other type of settings.

## Results

**Figure 1:**
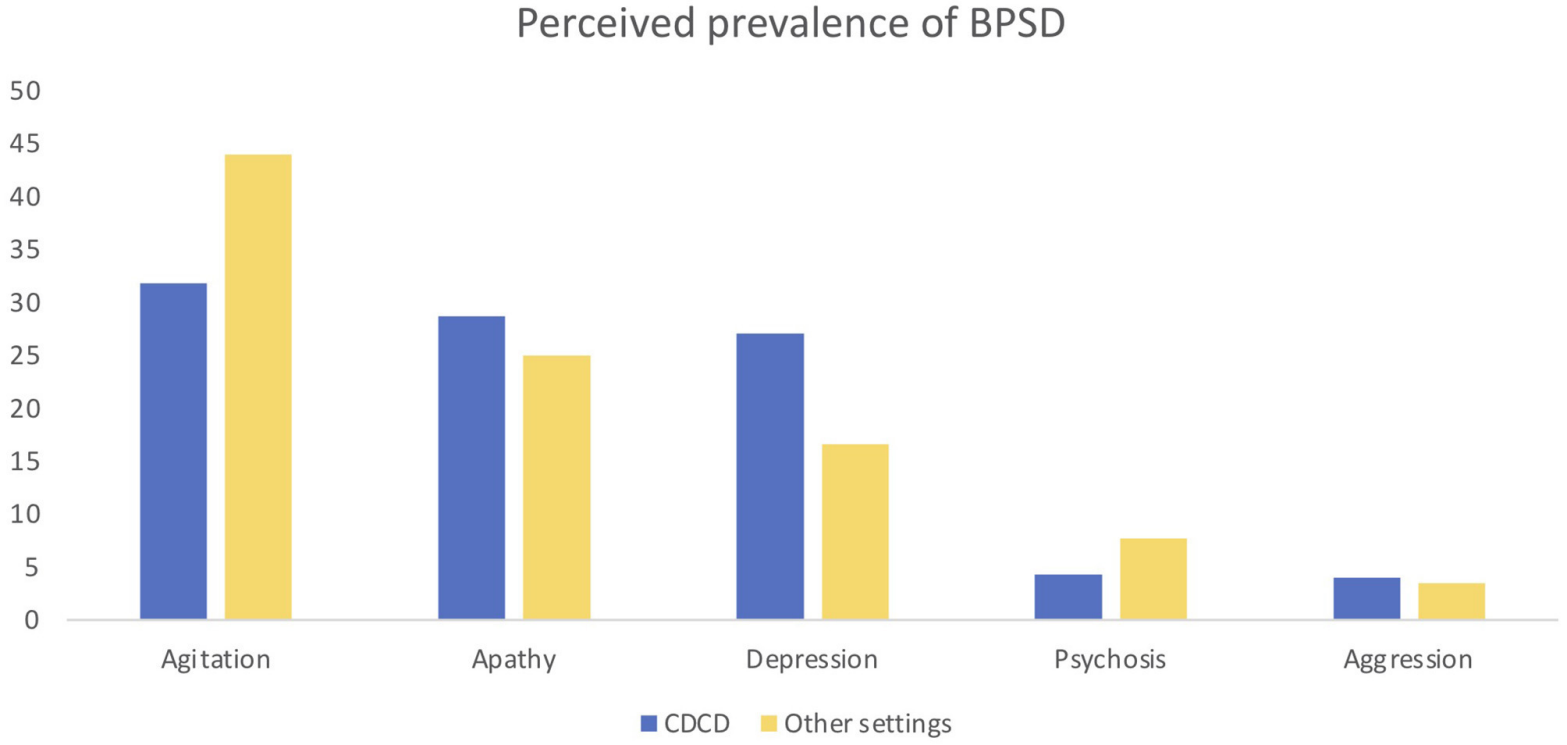
Answer differences between CDCD and non-CDCD setting: perceived prevalence of BPSD.

**Figure 2:**
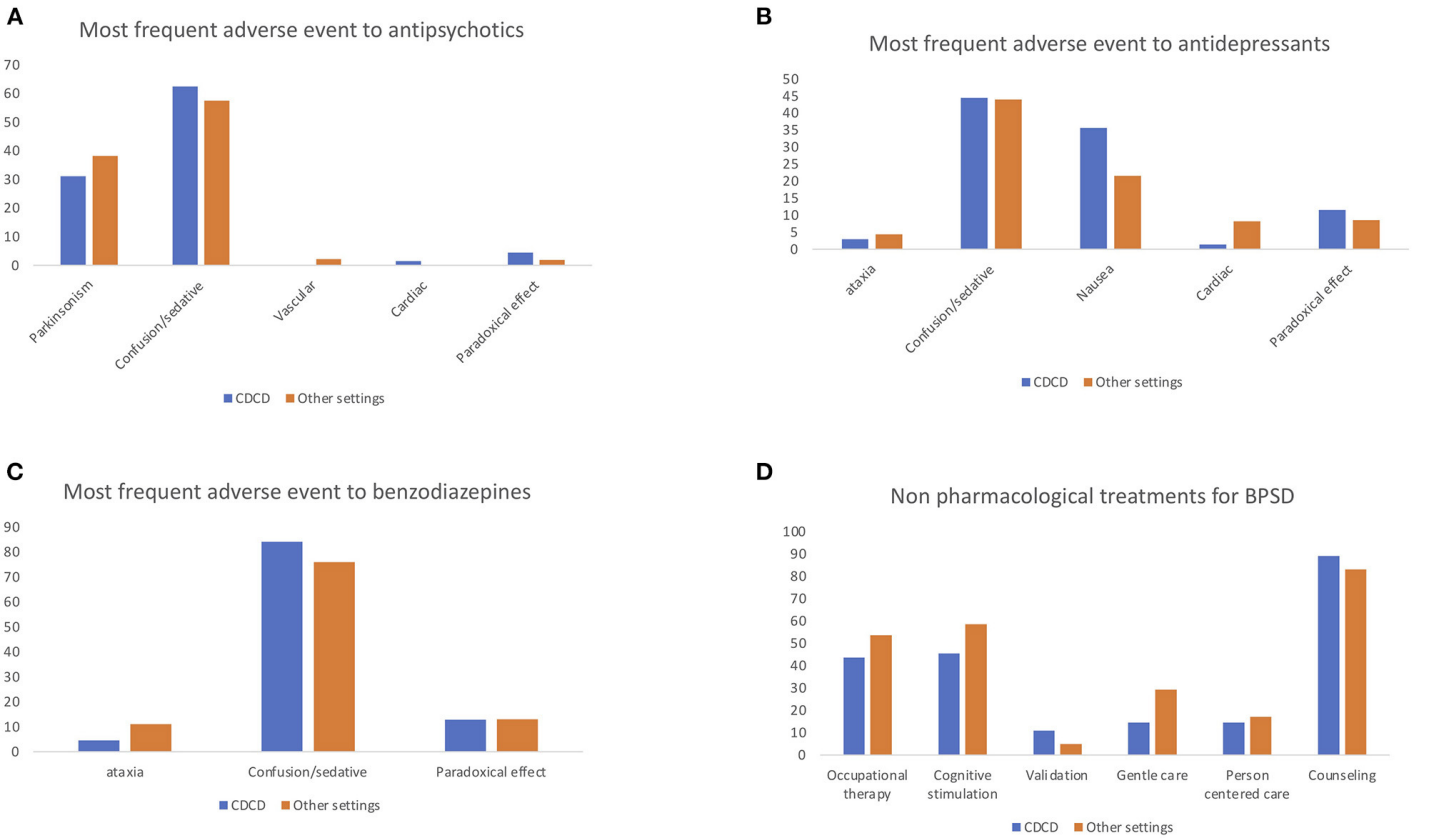
Answer differences between CDCD and non-CDCD setting: perceived most frequent side effects of antipsychotics **(A)**, antidepressants **(B)**, benzodiazepines **(C)**, and use of non-pharmacological approach to treat BPSD **(D)**.

One-hundred and thirty-six specialists participated in this study. Seventy-nine participants worked in a CDCD and 57 in other settings (e.g., general hospital wards, long-term care facilities, public outpatient services). The perceived frequency of BPSD was 74% and the importance of these symptoms was judged to be 88 over 100. BPSD are evaluated by means of a caregiver interview for 97% or by clinical assessment for 96.3%; dedicated tools are used by 72% of participants. Eighty-one percent use the complete NPI, 11.2% use NPI-questionnaire (NPI-Q), 1.6% use the Informant-based Behavioral Pathology in Alzheimer's Disease Rating Scale (BEHAVE-AD), and 6% other scales. The responders reported as most frequent symptoms agitation (37%), apathy (27.2%), and depression (22.5%) followed by psychosis (5.7%) and aggression (3.8%). Eighty-one percent of responders used drugs to counteract apathy, specifically antidepressants (SSRI 62.2% and SNRI 17.7%), but also dopaminergic drugs. For psychosis the first-choice treatments were atypical antipsychotics (83.3%), followed by typical antipsychotic (8.9%), and antidepressants (4.8%). For agitation, atypical antipsychotics were the first-choice treatment in 64% of cases and antidepressants in 16.1%. For aggression, the far most used drugs were atypical antipsychotics (82.9%); as a second line 36.6% used typical antipsychotics and 16.9% antidepressants. For depression most participants used antidepressants (85.2%); less frequent was the use of atypical antipsychotics (10.2%). For anxiety, 55.2% used antidepressants in the first instance, almost 17.9% use atypical antipsychotics, and 16.9% benzodiazepines, which were the second choice in 28.3% of the cases. For sleep and eating disorders, 62.9% and 60.4% of participants, respectively, used non pharmacological treatments as first choice interventions. For BPSD treatment 78.1% of the responders preferred a monotherapy and 15.3% a combination of treatments. In their opinion, 64.2% of the responders reported that acetyl-cholinesterase inhibitors (ACHeI) are effective for BPSD treatment and 66.6% reported the same for memantine. The most common reported side effects with antipsychotics were sedation/confusion (59.8%) and parkinsonism (34.6%). Interestingly, even if almost all responders were medical doctors and not psychologists, non-pharmacological approaches were reported to be used to treat BPSD, including caregiver counseling and education (86.4%), cognitive stimulation (51%), occupational therapy (47.9%), and gentle and person-centered care (20.8%). Blood tests are the most frequent exams to investigate BPSD (84.04%); neuroimaging such as CT and MRI is used in 60 and 64% of the centers. However, other exams are also used to this aim: 60% of participants use EEG, 29.7% FDG-PET, and 21.2% CSF analysis. Most responders expressed their interest in developing a new specific scale to evaluate BPSD (93.4%) and in specific biomarkers for BPSD (87.6%). Some differences emerged comparing the responses from CDCD and those from other care settings, specifically in BPSD treatment and reported treatments side effects. See Tables 1, 2 and Figures 1, 2 on this point.

**Table 1:** Answer differences in CDCD vs. non-CDCD setting; values ae expressed as percentages.

	**CDCD**	**NON-CDCD**
	**(*n* = 79)**	**(*n* = 57)**
How frequently do you observe BPSD?	73	75
How important are BPSD in your clinical practice?	88	87
**How do you evaluate BPSD?**		
Clinical evaluation	97.4	94.8
Information by caregivers	97.4	96.5
Dedicated tools	75.6	68.9
**Which assessment tool do you use?**		
NPI	80.2	82.7
NPI-Q	15.07	5.8
BEHAVE-AD	1.3	1.9
Others	2.7	9.6
**Which BPSD is more frequent (1st place)?**		
Agitation	31.8	44
Apathy	28.7	25
Depression	27.1	16.6
Psychosis	4.3	7.7
Aggression	4	3.5
**Do you usually treat apathy?**		
Yes	87.3	73.6
No	12.6	26.3
**Do you think AChEIs are effective for BPSD?**		
Yes	66.7	62.5
No	33.3	37.5
**Do you think memantine is effective for BPSD?**		
Yes	70.9	60.4
No	29.1	39.6
**What are the most frequent adverse event to antipsychotics?**		
Parkinsonism	31.1	38.2
Confusion/sedation	62.5	57.4
Vascular	0	2.1
Cardiac	1.4	0
Paradoxical effect	4.4	1.9
**What are the most frequent adverse event to antidepressants?**		
Ataxia	3	4.4
Confusion/sedation	44.4	44
Nausea	35.6	21.6
Cardiac	1.5	8.2
Paradoxical effect	11.6	8.5
**What are the most frequent adverse event to benzodiazepines?**		
Ataxia	4.6	11.1
Confusion/sedation	84	75.9
Paradoxical effect	12.9	13
**Do you usually prescribe drugs for BPSD in monotherapy or in polytherapy?**		
Monotherapy	84.8	68.4
Polytherapy	8.6	24.5
**Do you use non-pharmacological treatments for BPSD?**		
Yes	65.9	65.5
No	34.1	34.5
**Which non-pharmacological treatments do you use?**		
Occupational therapy	43.6	53.7
Cognitive stimulation	45.5	58.5
Validation	10.9	4.9
Gentle care	14.6	29.3
Person centered care	14.6	17.1
Counseling	89.1	82.9
**Do you perform diagnostic exams specific for BPSD?**		
Yes	27.9	21.4
No	34.2	35.7
**What procedures do you perform for BPSD?**		
MRI	61.1	59
CT	66.7	61.5
FDG-PET	31.5	28.2
EEG	61.1	59
Other functional neuroimaging	13	7.7
Blood exams	85.2	82.1
Lumbar puncture	20.4	23.1
**Would you be interested in clinical scale specific for BPSD?**		
Yes	94.9	91.4
No	5.1	8.6
**Would you be interested in biomarkers specific for BPSD?**		
Yes	91.1	82.8
No	8.9	17.2

**Table 2:** A comparison of first choice treatment options for neuropsychiatric symptoms between centers for cognitive disorders and dementia (CDCD) and other settings.

		**Anti-depressants**	**Atypical antipsychotics**	**Typical antipsychotics**	**Benzo-diazepines**	**Anti-epileptics**	**Dopaminergics**	**Non-pharmacological interventions**
Aggression	CDCD	1.5	***83.7***	6.9	2.9	2.9	-	-
	Others	4	***81.5***	8.2	2	4	-	-
Agitation	CDCD	15.1	***64***	5.5	1.4	8.2	-	-
	Others	17.7	***64.2***	8	1.9	10.2	-	-
Anxiety	CDCD	***57.5***	14.5	3.1	18.3	18.4	-	-
	Others	***51***	22.6	4.2	15.4	8.2	-	-
Apathy	CDCD	***92.2***	-	-	-	-	4.5	-
	Others	***85.3***	-	-	-	-	9.1	-
Depression	CDCD	***86.7***	13	1.5	-	-	-	-
	Others	***83***	6.4	2	4.4	2.1	-	-
Nutrition disorders	CDCD	14.7	16.2	3.1	1.5	-	-	***61.6***
	Others	18.9	18.4	-	0	-	-	***60***
Psychosis	CDCD	5.6	***84.4***	8.5	-	-	-	*-*
	Others	3.9	***81.8***	9.6	1.9	6	-	*-*
Sleep disorders	CDCD	12.6	13	1.5	9.9	-	-	***68.1***
	Others	11.3	23.1	0	13.2	-	-	***59.6***

*Data are expressed as percentages (CDCD: n. 79; other settings: n. 57). Bold values indicate the first choices*.

## Discussion

In this study, we present the results from a survey involving a large number of Italian specialists in the field of dementia concerning the general recognition, management, and opinions on BPSD.

Overall, the responders perceive the importance of BPSD and most of them assess these symptoms through a caregiver inquiry and dedicated tools. The most reported symptoms were agitation, apathy, and depression. Indeed, apathy is described to be the most common BPSD in dementia (21) and often occurs in early disease stage, whereas agitation is most common in later stages: it is probable that this kind of occurrence influences the subjective perception of these two symptoms, making them the most frequently perceived. Moreover, apathy and depression have been found to be predictive of MCI conversion to AD (22) and they can appear in the prodromal stage of dementia. Nevertheless, the perceived prevalence of these symptoms is lower than reported in the literature: thus, it would be important to improve the knowledge and awareness of BPSD among specialists working in this field. Although there are no specific recommendations for treatment of apathy, and it is well-known that apathy is different from depression (23), and most of the centers prescribe antidepressants to treat this symptom. It would be useful to investigate whether this strategy has some efficacy evidence in clinical practice, above all when we consider that antidepressants, and especially SSRI, can cause emotional blunting (24). Some responders prescribe dopaminergic treatments as well: this kind of therapy could have a more solid scientific basis (25). AChEIs and memantine were generally considered effective in the treatment of BPSD. In fact, it is well-known that AChEIs reduce psychotic symptoms in Lewy Body Dementia (26), but an effect on BPSD has been also advocated in AD (27). Even if medical agencies have reduced the impact of these medications on BPSD highlighting a small cost efficacy ratio, a survey on the management of BPSD and prescribing practices in the UK found that AChEIs were considered the second most appropriate pharmacological choice for psychosis after quetiapine (28), and recent studies have shown the effects of combination therapy on reducing dis-inhibition symptoms (29) and on improving all NPI domains except euphoria and apathy (30). For the control of aggression, most responders use atypical and typical antipsychotics as their first choice and, as a second line, antidepressants as well. Agitation and aggression appear to be symptoms difficult to treat, with physicians attempting different pharmacological classes to reduce symptoms. For anxiety, most used drugs were antidepressants followed by atypical antipsychotics and benzodiazepines. Some choices can appear unconventional, such as the use of antipsychotics as the first line for anxiety, but they can be explained by the clinician's attempt to avoid the use of benzodiazepines in dementia patients for their well-known side effects such as confusion and sedation. Moreover, anxiety in dementia can often be the epiphenomenon of restlessness or unmotivated fears or even psychotic beliefs.

It is worth noting that most of the centers apply non-pharmacological treatments for BPSD, especially caregiver counseling and education, and cognitive stimulation as well. Italian specialists on dementia seem therefore to be aware that most guidelines recommend a non-pharmacological approach as the first-line treatment for BPSD, even if there is no consensus on the best technique to be used (18).

When the responses to the survey were analyzed comparing the different settings, and in particular when comparing CDCD and other settings, some differences in BPSD perception, treatment options, and observed side effect were found. First, agitation was perceived as most common in other settings compared to CDCD, while apathy and depression were perceived as less frequent. Moreover, apathy was treated more frequently in CDCD than in the other settings, and the use of serotonin and norepinephrine reuptake inhibitors (SNRI) to this aim was higher than in the other settings. In anxiety treatment the atypical antipsychotics were more used in other clinical settings than in CDCD. For sleep disorders, specialists working in CDCD tend to use non-pharmacological treatment more than in other settings, while specialists working in other settings more often use atypical antipsychotics. The variability related to the work setting is also reflected in reported side effects. Atypical antipsychotics were reported to cause parkinsonism more frequently in other settings than in CDCD. Benzodiazepines were reported to produce ataxia more frequently in other settings then in CDCD. These data could depend on different possible factors: in a general hospital, where an acute confusional state is often superposed to dementia and where the care setting is not adapted to the special needs of people with dementia, larger doses of antipsychotics and benzodiazepines could be used than in the specialized CDCD setting. In day care centers, there are many people with dementia in the moderate-severe phase, when agitation, aggression, and sleep disorders are more frequent. Therefore, neuroleptics and benzodiazepines could be used more frequently and at larger dosage. Non-pharmacological therapy is used in both settings but, while specialists working in CDCD mainly prefer the caregiver counseling, in other settings cognitive stimulation and occupational therapy are used more often. This difference could be due to organization difficulties: CDCD are ambulatory patients services, while day care centers or long-term facilities are residential settings where it is easier to organize formal and/or group therapies for patients. Another difference was the interest in the new biomarker development which was more conspicuous in CDCD than in other settings. This could be due to the fact that CDCD are the main reference in Italy for diagnosis and treatment of dementia: biomarker development could facilitate these tasks. Biomarkers could include structural and functional neuroimaging correlates or cerebro-spinal fluids (CSF) biomarkers. Findings on neural correlates associated with neuropsychiatric syndromes have been summarized in a review by Rosenberg et al. (31). The authors reported that structural and functional alterations associated with agitation may be related to alterations in the regions involved by the AD core pathology and also to brain regions associated with emotional regulation (amygdala) and salience (insula), while apathy was found to be associated with structural and functional alterations in regions such as the anterior cingulate cortex and amygdala. Delusions were instead associated with alterations in frontal and temporal regions (31–35). Moreover, research on CSF biomarkers suggest that elevated CSF tau might be associated with agitation (36) and with psychosis in AD (37, 38). Improving research on BPSD biomarkers could help to better characterize these symptoms and could help to develop new treatment strategies.

The variability in the responses about BPSD treatment can obviously be explained also by the absence of clear guidelines, in addition to differences in patients' characteristics and clinical practice based on subjective experience. However, some general principles are generally shared by Italian specialists, such as using antipsychotics in the lowest dose sufficient to control symptoms, with close monitoring for adverse effects.

The fact that most of the centers involved in the survey use NPI to detect neuropsychiatric symptoms deserves considerations. Indeed, NPI has some limitations. Specifically, it does not allow investigation of the detailed phenomenology of each BPSD, nor does it investigate symptoms according to BPSD clusters. Moreover, the NPI questions refer just to a specific time period (previous 4 weeks). However, according to the hypothesis that BPSD may represent a patient's trait and not only a patient's state, information about patient's BPSD history can be missed. A further important aspect is that the NPI is administered to caregivers and it can be influenced by the personal caregiver's perception and burden. Thus, NPI lacks a direct observation of the patients by the clinicians. It would be helpful to validate and standardize a scale to investigate BPSD comprising both caregiver's perception and patient's observation, which might also be useful to detect drugs effects in clinical trials targeting BPSD.

The differences outlined by our survey underline that for clinicians the choice of the right drug for a specific BPSD and therapy-tailoring is challenging.

A study that applied a standard methodology such as the Appraisal of Guidelines Research and Evaluation (39), an instrument for the quality assessment of clinical practice guidelines, compared the existing guidelines for BPSD treatment with the aim to detect recommendations with great impact. The authors reported that, while some agreement exists on the use as first choice of non-pharmacological treatment, and on the antipsychotics, overall research in BPSD management is lacking, and they highlighted the need of formulating specific guidelines (18).

This study has some limitations. Since the survey investigated many aspects of BPSD management, to warrant a high proportion of responses, it was not possible to include too many or too detailed questions, such as drug dosages, treatment durations, or specific tools used to assess BPSD in different types of dementia or disease stage. Future studies are needed to investigate these aspects. Another limitation is that some care settings were in fact excluded or underrepresented such as the homecare, geriatric wards, and long-term care facilities (as there were more neurologists than geriatricians among the responders). It would be also important to investigate BPSD perception and management in context different from those specialized in dementia field, such as primary care. Since the survey reported data based on clinicians' impressions, quantitative data about BPSD management were not collected. Moreover, it was not possible to investigate in more detail non-pharmacological treatments, although many of the centers reported them as first choice for BPSD. Further studies focusing on these aspects are also warranted.

Our results suggest that producing guidelines focusing on management and pharmacological treatment of BPSD is a major need. Specific practice recommendations in existing dementia guidelines should be formulated to clarify for each BPSD which drug should be used as first line and second line at which dosage and possibly for how long. Moreover, it would be important to specify if some drugs are more effective in specific forms of dementia, and whether it is better to use them in monotherapy or in polytherapy. Specific guidelines could help in an effort to harmonize BPSD management and provide clear recommendation for clinicians in the dementia field.

## Data Availability Statement

The original contributions presented in the study are included in the article/supplementary materials, further inquiries can be directed to the corresponding author.

## Participants to the BPSD Study Group

Margherita Alberoni, Serena Amici, Andrea Arighi, Francesca Baglio, Federica Barocco, Amalia Cecilia Bruni, Giuseppe Bruno, Annachiara Cagnin, Elena Calabrese, Antonio Callari, Marco Canevelli, Rosanna Colao, Matteo Cotta Ramusino, Eduardo Cumbo, Chiara Cupidi, Alfredo Costa, Sabrina Curcio, Chiara Cutaia, Carlo de Lena, Mario Tommaso dell'Osa, Babette Dijk, Francesco Di Lorenzo, Maria Grazia Di Maggio, Andrea Francescani, Francesca Frangipane, Valeria Isella, Claudio Ivaldi, Sebastiano Lorusso, Antonina Luca, Giuseppe Magnani, Luigi Giovanni Manfredi, Michele Maniscalco, Lorenzo Marchese, Michela Marcon, Alessandra Marcone, Maria Giuseppina Mascia, Antonio Milia, Concetta Mina, Cristina Moglia, Flavio Mariano Nobili, Giulia Perini, Patrizia Perrone, Giuseppina Pilia, Federico Pozzi, Gianfranco Puccio, Francesca Saibene, Ermanno Matteo Soave, Elena Sinforiani, Micaela Sepe Monti, Michelangelo Stanzani Maserati, Andrea Stracciari, Gloria Tognoni, Marco Vista.

## Author Contributions

FD'A, LT, MZ, SP, and EF: conceptualization and writing—review and editing. FD'A, LT, and EF: methodology and formal analysis. Sindem BPSD Study Group: resources. FD'A: writing—original draft preparation. EF: supervision. All authors contributed to the article and approved the submitted version.

## Funding

This work was supported by the Italian Ministry of Health (Ricerca Corrente).

## Conflict of Interest

EF was employed by IRCCS Don Gnocchi Foundation. The remaining authors declare that the research was conducted in the absence of any commercial or financial relationships that could be construed as a potential conflict of interest.

## Publisher's Note

All claims expressed in this article are solely those of the authors and do not necessarily represent those of their affiliated organizations, or those of the publisher, the editors and the reviewers. Any product that may be evaluated in this article, or claim that may be made by its manufacturer, is not guaranteed or endorsed by the publisher.
